# Gut and Lung Microbiota in Preterm Infants: Immunological Modulation and Implication in Neonatal Outcomes

**DOI:** 10.3389/fimmu.2019.02910

**Published:** 2019-12-12

**Authors:** Chiara Tirone, Lucilla Pezza, Angela Paladini, Milena Tana, Claudia Aurilia, Alessandra Lio, Silvia D'Ippolito, Chiara Tersigni, Brunella Posteraro, Maurizio Sanguinetti, Nicoletta Di Simone, Giovanni Vento

**Affiliations:** ^1^Fondazione Policlinico Universitario A. Gemelli IRCCS, U.O.C. di Neonatologia, Dipartimento di Scienze della Salute della Donna, del Bambino e di Sanità Pubblica, Rome, Italy; ^2^Università Cattolica del Sacro Cuore, Istituto di Clinica Pediatrica, Rome, Italy; ^3^Fondazione Policlinico Universitario A. Gemelli IRCCS, U.O.C. di Ostetricia e Patologia Ostetrica, Dipartimento di Scienze della Salute della Donna, del Bambino e di Sanità Pubblica, Rome, Italy; ^4^Università Cattolica del Sacro Cuore, Istituto di Clinica Ostetrica e Ginecologica, Rome, Italy; ^5^Fondazione Policlinico Universitario A. Gemelli IRCCS, Dipartimento di Scienze di Laboratorio e Infettivologiche, Rome, Italy; ^6^Istituto di Microbiologia, Università Cattolica del Sacro Cuore, Rome, Italy

**Keywords:** gut microbiota, lung microbiota, preterm infants' outcomes, gut-lung axis, late-onset sepsis, necrotizing enterocolitis, growth impairment, bronchopulmonary dysplasia

## Abstract

In recent years, an aberrant gastrointestinal colonization has been found to be associated with an higher risk for postnatal sepsis, necrotizing enterocolitis (NEC) and growth impairment in preterm infants. As a consequence, the reasons of intestinal dysbiosis in this population of newborns have increasingly become an object of interest. The presence of a link between the gut and lung microbiome's development (gut-lung axis) is emerging, and more data show as a gut-brain cross talking mediated by an inflammatory milieu, may affect the immunity system and influence neonatal outcomes. A revision of the studies which examined gut and lung microbiota in preterm infants and a qualitative analysis of data about characteristic patterns and related outcomes in terms of risk of growing impairment, Necrotizing Enterocolitis (NEC), Bronchopulmonary Dysplasia (BPD), and sepsis have been performed. Microbiota take part in the establishment of the gut barrier and many data suggest its immune-modulator role. Furthermore, the development of the gut and lung microbiome (gut-lung axis) appear to be connected and able to lead to abnormal inflammatory responses which have a key role in the pathogenesis of BPD. Dysbiosis and the gut predominance of facultative anaerobes appear to be crucial to the pathogenesis and subsequently to the prevention of such diseases.

## Introduction

All of the microorganisms that inhabit the human body constitutes the so-called human microbiota. The Human Microbiota Project was launched in 2008 to deepen our understanding of how the microbiome (the whole set of microorganisms, their genomes and the environmental conditions) influences human health and diseases. 16S ribosomal RNA (16S rRNA) sequencing allows characterizing the complexity of the microbial population to study whether there is an “healthy microbiota” and potential implications of different patterns (1).

The importance of gut microbiome is due to the role it plays as a major interface to the external environment: it contemporarily protects against pathogens and toxins while housing beneficial commensal bacteria which are pivotal to maintain homeostasis, support digestion, protect from injury, regulate intestinal immune function (2).

Some studies (3, 4) show that at birth infants are nearly sterile but they subsequently acquire microbial colonists. This process progresses in the first 2–3 years of life, until reaching an “adult-like state.” In at term newborns this evolution appears to be driven by nutritional, immunological, hormonal and prebiotic effect of maternal milk (5).

It is difficult to identify a “healthy” microbiota of a population such that of preterm infants. The preterm birth is a non-physiological condition, exposed to many early life clinical factors that alter the normal colonists acquisition process (6, 7).

The early life can be defined as a “critical window” during which the occurrence of dysbiosis can impact the health of preterm infants, especially influencing the developing immune systems (8–10).

Moreover, Olm et al. (11) underlined as our understanding of the habitat range and subpopulation complexity of founding strains is impaired by methodological limitations. These authors compared the *in situ* bacterial growth rates of multiple body sites by using metagenomics to reconstruct the genomes of strains that colonized the skin, mouth, and gut of two hospitalized premature infants. The results show an overlap of strains across body sites and imply that the premature infant microbiome is characterized by a low total microbial diversity of the early community when compared to full-term infants.

Anyway, in recent years, many studies regarding the features of gut and lung microbiota of preterm infants have followed.

### Gut Microbiota in Preterm Infants

Altought newborns' gut was thought to be sterile and commensal microbes only acquired after birth, recently growing evidences seem to suggest that non-sterile intrauterine conditions could be the origin of this acquisition: Aagard et al. performed a whole-genome shotgun metagenomic study of placental specimens. Their results showed a unique placental microbial flora that comprises non-pathogenic commensal microbes belonging to the *Tenericutes, Firmicutes, Bacteroidetes, Proteobacteria*, and *Fusobacteria* phyla (12); Collado et al. reported that the amniotic fluid hosts a distinct microbial community characterized by a predominance of *Proteobacteria* (13).

Moreover, a recent microbial profiling study based on 16S rRNA sequencing shows that regardless of the delivery mode, the microbial population in the meconium is influenced by that in the correspondent maternal placenta (14).

Vaginally delivered newborns directly come into contact with vaginal microbial population and their fecal microbiota is dominated by *Prevotella* and *Lactobacillus* (15, 16) while newborns delivered by cesarean section are more likely to have a microbiota dominated by microbes derived from maternal skin, hospital environment and even hospital staff such as *Corynebacterium, Staphilococcus* and *Propionibacterium* spp. (6, 16–18). It is also well-known that neonatal gut microbiota is strongly influenced by food intake: stools of breast-fed are richer of *Lactobacilli* and *Bifidobacteria* and poorer of potential pathogens than formula-fed newborns whose stools contain a more diverse microbial flora with a prevalence of *Bacteroides, Staphilococci, Clostridia, Enterococci, Enterobacteria* (19–22).

Gestational age is another pivotal influencing factor, for different orders of reasons: preterm infants have immature gastrointestinal and immune systems; they are precociously exposed to extensive use of antibiotics and often long term hospitalized; they need mechanical ventilation and usually receive parenteral nutrition. Each one of these conditions may produce irreversible change into the natural process of colonization and development of the gut microbiota (23).

Particularly, in these newborns anaerobic colonization is delayed and their stools host higher levels of *Enterobacteriaceae, Enterococcus*, and opportunistic pathogens if compared with term newborns (24–29).

Recently, Tauchi and colleagues described a delayed *Bifidobacteriaceae* colonization, underlining the role of *Bifidobacterium* as probiotic to induce “normal” infant microbiota. Their data also suggest a longer predominance of *Staphilococcacae* in preterm infants with an increased risk of potentially pathogenetic methicillin resistant *Staphylococcus aureus* colonization in NICU infants (30).

Korpela et al. (31) performed a study on fecal samples from 45 preterm infants, in order to identify a pattern of development of the intestinal microbiota. Four phases were identified with a pattern of progression to a *Bifidobacterium*-dominated composition typical of the full-term non-hospitalized newborns. This normal-like microbiota development correlated with post-menstrual age, was achieved also in cesarean-delivery newborns and was favorited by administration of breast milk. Moreover, these authors observed as among the extremely premature infants the overgrowth of *Enterococcus* spp. inhibit the normal succession, while antibiotics administration causes temporary changes in intestinal microbiota composition that subsequently recovers after few days.

Table 1 summarizes different categories of typical gut microbial pattern that can be found in healthy newborns. The principal variables that can affect the rising abundance of a population over another are reported and it is clearly showed as there's not a single typical healthy microbiota, but gut microbiota is rather a spectrum, result of all the combination of variables that coexist in an infant.

**Table 1:** Summary of different categories of typical gut microbial pattern that can be found in healthy newborns.

	**Gut characteristic bacterial populations in healthy newborns**
Term neonates	*Lactobacillus, Bifidobacterium*
Preterm neonates	*Enterobacteriaceae, Veillonella, Enterococcus, Staphylococcus*
Breast feed	*Lactobacillus, Bifidobacterium*
Formula feed	*Bacteroides, Staphylococcus, Clostridia, Enterococcus, Enterobacteria*
Vaginally delivered	*Prevotella, Lactobacillus*
C-section	*Corynebacterium, Staphylococcus, Propionibacterium*

*We report the principal variables that can affect the rising abundance of a population over another: the correspondence may concern either a genus (such as Staphylococcus), either a family (such as Enterobacteriaceae). It clearly shows that there's not a single typical healthy microbiota, but gut microbiota is rather a spectrum, result of all the combination of variables that coexist in an infant*.

### Lung Microbiota in Preterm Infants

Lungs have historically been considered sterile in healthy people so that they were initially omitted from the priority organ system studied by the Human Microbiome Project; subsequent culture independent studies demonstrated that lungs host diverse communities of bacteria and the interest in this new field rapidly grew since the linkage of different microbiomes with specific respiratory diseases was observed (32).

Sampling from lungs and distal airways represents a major challenge in neonatal population because of low biomass with bacterial loads close to the detection limit of the assays and for the risk of contamination from upper airways (33).

Timing of colonization is debated. Mourani and colleagues noted that only 2 of 10 tracheal aspirates of intubated preterm infants contained detectable bacterial DNA in the first 72 h of life, whereas all samples from the same newborns were positive at day 7 (34). In contrast, Lohmann et al. reported that bacterial DNA was detectable in all tracheal aspirates taken immediately after intubation at day 1 of life from 25 preterm newborns (35) so that it appears that colonization of the airways begins very early or even before the delivery.

The development of lung microbiota in the preterm infant can be affected by several factors: first of all, maternal chorioamnionitis (which is a pivotal cause of premature birth); then exposure to mechanical ventilation, antibiotics, NICU environmental microbial population, feeding, gut microbiota composition.

An aberrant gastrointestinal colonization has been found to be associated with a higher risk for postnatal sepsis, necrotizing enterocolitis (NEC) and growth impairment in preterm infants. As a consequence, the reasons of intestinal dysbiosis in this population of newborns have increasingly become an object of interest.

Moreover, recently, there's a rising attention to the so-called “gut-lung axis.” The presence of a link between the gut and lung microbiome's development is emerging. Gut and lung microbiota are known to be pivotal in the education of host immune system, as reported by Dang et al. (36). Both microbes and their products participate to this complex interaction. The intricate relationship between gut and lung microbiota is testified by the bidirectional association of gut dysbiosis with lung disease: Kalliomaki et al. (37) already observed an increased abundance of Clostridia and reduced Bifidobacteria in the gut of infants who developed early life asthma; the review by Dang (36) also shows evidences of gastrointestinal perturbations in patients with chronic lung disease. The “mucosal response theory “reported by Gallacher and Kotecha (33) implies that dendritic cells (DCs) in the intestine come into contact with the resident microbial population; it generates signals that result in phenotypic changes in the DCs which migrate to the mesenteric lymph nodes where they stimulate the production of regulatory cytokines such as IL-10, TGF-beta, INF-gamma, and IL-6. In the mucosa-associated lymphoid tissue, DCs present the bacterial derived antigens to T-cells, leading to their activation. T cells then acquire homing molecules like chemokine receptor 4 (CCR-4) and chemokine receptor 6 (CCR-6). Moreover, the activated T cells can reach the respiratory mucosa where they promote protective and anti-inflammatory responses. Other mechanisms of interaction have been highlighted: Short Chain Fatty Acids (SCFA), gut microbioma derived metabolites, have a kay role; they reach the blood stream and come to the bone marrow were they promote hematopoiesis stimulating the differentiation of the Hematopoietic Stem Cells (HSCs) toward an anti-inflammatory milieu which migrates in the airways (36).

More data show that an impairment of the lung microbiota could be implicated in the pathogenesis of BPD.

These new evidences are increasingly interesting, since the manipulation of maternal microbiota (during or even before pregnancy) might influence the pregnancy outcome and the fetal/infantile health.

## Objectives

We evaluated the studies that examined gut and lung microbiota in preterm infants and summarized emerging evidences. Our objective was to perform a qualitative analysis of data about characteristic patterns leading to dysbiosis in different body sites and to evidence the actual knowledge of the role of dysbiosis on preterm infants' outcomes such as growing impairment, Necrotizing Enterocolitis (NEC), Bronchopulmonary Dysplasia (BPD) and sepsis.

## Materials and Methods

In order to identify papers considered in this qualitative review, we performed a literature search on PubMED. The research has been restricted to papers in English language. We limited the search by applying the filter of age “infants” and used the following search terms and logic: “preterm infants microbiota,” “gastrointestinal microbiome *AND* Necrotizing Enterocolitis *OR* NEC,” “breastfeeding *AND* enteral nutrition *AND* Necrotizing Enterocolitis *OR* NEC,” “microbiota *AND* growth retardation,” “intestinal microbiota *AND* weight gain,” “intestinal microbiota *AND* growth,” “gut microbiota *AND* extrauterine growth restriction,” “preterm infants microbiota *AND* late onset sepsis *OR* LOS,” “microbiota *OR* microbiome *OR* bacteria *OR* antibiotics *OR* gut *AND* lung *OR* airway *OR* BPD *OR* Bronchopulmonary Dysplasia,” and “gut-lung axis.” No limit about year of publication has been set, and the final search is updated to July 2019.

To identify any articles that may have been missed during the literature search, also reference lists of candidate articles have been carefully checked.

## Discussion

### The Preterm Gut Microbiota and Growing Impairment

Postnatal growth failure is a frequent adverse outcome in preterm infants that occurs during a critical developmental period. The study of the extrauterine growth rates of extremely preterm (EPT) infants (i.e., birth gestational age ≤27 weeks) show that approximately half of those infants remain below the 10th percentile in weight of the reference *in utero* growth rates, at the time of neonatal intensive care unit (NICU) discharge (38, 39).

Gut microbiota, gastrointestinal tract, and immune system maturation appear to be affected by prematurity and nutrition. Particularly, the gut microbiota, with its distinct metabolic capacities, plays a role in the metabolism of dietary components, which appear to be indispensable for the host. Many factors are involved in the variation of gut microbiota in preterm infants, such as the hospital environment of the NICU and its associated common clinical practices and feeding regimens, with subsequent direct and indirect interference with energy harvest and storage, and thereby with weight gain (40–42). Indeed, the gut microbiota, with its complementary metabolic capacity to human gastrointestinal enzymes, is able to provide the host with nutrients and energy otherwise unavailable (43). It also interferes with the host body weight management (40, 44–46) by producing metabolites and by affecting the harvest, storage, and expenditure of energy from food components (43, 47).

The association between the gut microbiota, growth, and development in early life has been investigated by some studies in preterm infants (41). Of particular interest is the study of Grier et al. (42). These authors identified in preterm infants three phases (P1, P2, and P3) that correspond to the three states of the microbiota with distinct metabolic functions. Significant associations were found between nutrition, microbiota phase, and preterm infant growth (42). The microbiota analysis showed that P1 is associated to a low level of initial diversity with a predominance of facultative anaerobes. The transition out of P1, which could be identified with transition from meconium to normal postnatal stool, was characterized by increasing diversity and abundance of obligate anaerobes with a shift to fermentation-based metabolism in P3. A role of Paneth cells (PCs) AMPs in the modulation of the shift toward a community dominated by obligate anaerobes was suggested by the increase in the number of PC around a post-menstrual age (PMA) of 29 weeks, corresponding to the transition from P2 to P3. According to others studies (48, 49), *Bifidobacterium*, an Actinobacterium involved in the development and maintenance of the healthy infant gut microbiota, was significantly associated with lipid and protein intake in P3 as testified by its increased abundance with increased lipid in the diet and decreased abundance with greater amounts of protein. Moreover, an increased abundance of *Bifidobacterium* was also significantly associated with the use of corticosteroids and H2 receptor antagonists in P3. In addition, Proteobacteria resulted significantly associated with lipid intake, Firmicutes with protein and Actinobacteria, Proteobacteria, and Firmicutes with carbohydrates (42).

In contrast to these findings, in the study of Blankstad et al. (50), where the relationship between a fortified diet (with more energy, protein, fat, vitamin A, arachidonic acid and docosahexaenoic acid) and the intestinal microbiota development has been evaluated, *Bifidobacterium* was more represented among infants with standard nutrient supply and it was associated with an improved weight gain and, consequently, with overall better growth. Anyway, the authors postulated that the lower abundance of this bacterial genus in infants receiving the “fortified” nutrient supply could be explained as a consequence of a concomitant greater abundance of other microbes, and not necessarily less *Bifidobacterium* as a direct effect. Moreover, in this study (50), while the initial richness after birth did not appear to be influenced by nutrition, the maintenance of richness was observed only in the preterm infants who were fed with the fortified diet if compared with the standard nutrient supply. After the postnatal peak, all infants showed a decreased of microbial diversity, regardless of diet fortification.

Specific bacterial families and genera resulted to be associated with weight gain at 1 month of age also in the study of Arboleya et al. (41), with a correlation of growth rates and the levels of Enterobacteriaceae and Streptococcus at 2 days of age and of Bacteroides-group at 10 days of age. Moreover, some bacterial genera such as Staphylococcus and Enterococcus were negatively associated with weight gain, while Weissella was positively associated with weight gain in preterm infants (41). Concerning Weissella, there are interesting evidences that this genus could influence food digestion and energy harvest in infants (40, 43, 45). This aspect should be further explored, considering that lactobacilli, to which order Weissella belongs, are used as probiotics.

The results of a recent study (51), show that infants fed with the mother's own milk (MOM) had a greater abundance of *Bifidobacterium* and Bacteroides, each of that seems to be protecting against morbidities like NEC. Moreover, feeding primarily MOM was related to an increased gut microbiota diversity that had previously been related to healthier outcomes in VLBW infants and that resulted associated to a superior growth in comparison with infants fed with donor human milk.

Li et al. (52) demonstrated that preterm infants with growth failure have a distinct intestinal microbiome's profile when compared to infants with normal growth at postnatal days 1 and 28. At both time points, the sole highly abundant taxa in the EUGR group was the genus Parabacteroides, which is a gram-negative, anaerobic, non-spore forming genus. Some species of that genus turn out high quantities of acetic acid and propionic acid (53). Intestinal epithelial cells (IECs) can absorb these short fatty acids that can also enter the blood circulation, influencing the storage of sugar in muscle, liver, and fat. Also, the brain appears to be reached by acetic acid, with subsequent loss of appetite, and leading to reduced food intake (54). Thus, weight gain might relate to an excessive colonization of genus Parabacteroides in the intestinal tract of VLBW infants.

The study results of Yee et al. (55), complement previous data, showing an abundance of Proteobacteria in the preterm infants' microbiome (56, 57). These authors described a negative correlation between infant weight gain during hospitalization and the relative abundances of Klebsiella and Staphylococcus. Both of these taxa are associated with known pathogens so that their enrichment could be a sign of dysbiosis, finally associated with reduced infant weight and length gain.

A deficit in anabolic metabolism of glucose and other non-lipid energy source, resulting in a greater reliance on fatty acids to satisfy metabolic demands, has been proposed in preterm infants with growth failure from Young et al. (58) From their data emerged an alteration of the microbiota maturation characterized by low diversity, persistent dominance of Enterobacteriaceae, and a deficiency of strictly anaerobic taxa such as Veillonella in preterm infants with postnatal growth failure. These infants also demonstrated a “metabolic signature,” showing an increased lipolysis and fatty acid oxidation, with gain in multiple fatty acids, acylcarnitines, glycerol, and β-hydroxybutyric acid, that is distinctive of a fasted state.

Considering all the evidences reported (briefly summarized in Table 2), the knowledge of the mechanisms leading the infant microbiota to alter nutritional efficacy appears of great importance in the aim of predicting, preventing and treating growth failure in preterm infants.

**Table 2:** Summary of the changes in microbial resident population observed in the different pathologic conditions and the underlying pathogenetic mechanisms.

	**Change in microbial populations**	**Pathogenetic mechanisms involved**
BPD	Decreased abundance of *Lactobacillus*, reduction in the bacterial community turnover with increasing time from birth, decreased colonization by *Staphylococcus* in the first days after birth, increased colonization by *Ureaplasma*	A decreased abundance of protective species allows a relative overgrowth of potentially pathogen microbial populations; moreover, Ureaplasma-colonized newborns present peripheral blood leukocytosis and less severe RDS but early radiographic and histologic changes typical of BPD
EUGR	Low diversity, persistent dominance of *Enterobacteriaceae*, and a paucity of strictly anaerobic taxa including *Veillonella* compared to infants with appropriate growth. Relative abundance of *Klebsiella* and *Staphylococcus*. Increased rate of *Parabacteroides*.	Nutritional efficacy is dramatically affected by infant dysbiosis. In particular, *Parabacteroides* produce acetic and propionic acid that reach the blood circulation, affecting the storage of sugar in muscle, liver, and fat; acetic acid is also involved in reduced food intake due to loss of appetite.
LOS	Decreased bacterial diversity with a predominance of *Proteobacteria* and *Firmicutes*, whereas *Bifidobacteria* are usually found in healthy controls. A strong relation with *Fusobacteria* and *Tenericutes* has also been described.	Gut microbiota can translocate across a dysfunctional or immature intestinal barrier, overwhelm neonatal immune system and cause sepsis; moreover, intestinal dysbiosis is supposed to alter local and systemic immune function. Natural prebiotics, such as raffinose, inhibit the growth of potentially pathogenic bacteria and promote proliferation of *Bifidobacterium*.
NEC	Decreased bacterial diversity with increased rate of *Clostridia, Enterobacteriaceae*, and *Staphylococcus* are frequently observed; a predominance of *Proteobacteria* (such as *Escherichia coli* and *Klebsiella*) is considered strictly associated with the pathogenesis of the disease.	Antibiotics pressure and enteral feeding generate changes in gut microbiota which may lead to an overgrowth of potential pathogens; moreover, LPS of Gram Negative activates TLR-4 and premature gut reacts with an exaggerated cytokine mediated inflammatory response, which may be due to a deficient expression of inhibitors of the NFkB pathway.

*BPD, Bronchopulmonary Dysplasia; EUGR, Extra-Uterine Growth Restriction; LOS, Late-Onset sepsis; NEC, Necrotising Enterocolitis*.

### The Preterm Gut Microbiota and NEC

Necrotising EnteroColitis (NEC) is a major cause of mortality and morbidity in premature newborns; it affects 7% of newborns with birth weight <1500 g, of whom 20–30% dies (2). It is characterized by submucosal hemorrhage and oedema, neutrophilic infiltration of the intestinal wall, disruption of intestinal villus architecture and finally full thickness necrosis or intestinal wall perforation (59). Typically, the involved newborn presents with abdominal distension, bloody stools and feeding intolerance, while pneumatosis intestinalis and portal venous gas are characteristic abdominal radiological findings (60).

The pathogenesis of the disease is unclear but probably multifactorial: enteral feeding, intestinal ischemia and aberrant microbial colonization are the principal involved factors.

Initial exposition to microbes and their metabolic products is normal part of development with a largely unexplored effect on immune system. Breastfeeding is known to protect against NEC (61, 62): the reason is that breastfeeding facilitates colonization by a balanced commensal flora which contrasts bacterial overgrowth; instead formula feeding is associated with harmful gut bacterial proliferation (63). Preterm infants early come into contact with different environmental microbic species and are also precociously exposed to antibiotic therapies which have shown to reduce the diversity of infant microbiota; moreover, perturbations in the composition of infant microbiota may let pathogenic microbes prevail over commensal species (64).

A broad spectrum of microbes has been involved in the pathogenesis of NEC across multiple studies. According to recent evidences certain microbial population such as *Bifidobacteria* and *Lactobacilli* are protective, while *Clostridia, Enterobacteriaceae*, and *Staphylococcus* have been associated to the development of the disease (65).

Emerging data suggest that a predominance of *Proteobacteria* is strictly associated with NEC. Wang et al. (66) showed that infants in the NEC group have more than 50% of gut bacterial colonization represented by the *Gammaproteobacteria* genera such as *Cronobacter sakazakii, Klebsiella sp*., and *Escherichia coli*. This result was not found in the healthy newborns. The same pattern of gut microbiota has been reported by other authors (57, 67).

But in the stool of infants with NEC are often found also *coagulase-negative staphylococci* (*CoNS*) from the phylum *Firmicutes* (68) and *Clostridium* spp. with the associated toxin (69). In the past, this last microbe, that is part of the commensal microbiome of preterm infants, was associated with the progression of NEC (70, 71).

Concerning this aspect, it is known how bacterial products are recognized via Microbial Associated Molecular Patterns (MAMPs) by Pattern Recognition Receptors (PRRs) and in particular by the more studied Toll Like Receptor (TLRs) expressed on the intestinal mucosa: this interaction results in the activation of Nuclear Factor- kappa B (NFkB) and its inflammatory pathway and caspases. These propagate the apoptosis and induce the production of cytokines (INF-1, TNF-alfa, IL-1, IL-6, IL-8) via activation of transcription genes (72). TLRs are pivotal to maintain the equilibrium between adequate inflammatory response and homeostasis, performing crucial functions like regulation of cell proliferation and growth, antimicrobial agents secretion, and control of the barrier function (2).

After birth MAMPs start to interact with TLRs; moreover Lipopolysaccharide (LPS) of Gram Negative activates TLR-4 (73).

Premature gut reacts to the TLR-4 activation with a more accentuated cytokine mediated inflammatory response if compared to a full-term gut; IL-8 is especially involved in neutrophil chemotaxis increase and inflammation, leading in most severe cases to tissue injury and NEC (74, 75). This exaggerated inflammatory response may be due to a deficient expression of inhibitors of the NFkB pathway (76, 77). Commensal bacteria seem to have a beneficial role in the repression of the continuative stimulation occurring in the infant gut by blocking the degradation of NFkB inhibitors; they also contribute to the neonatal gut homeostasis generating a low-level stimulation of TLR-4 (78, 79) (Table 2).

Probably, a single pathogen entirely responsible for NEC will never be isolated, since there is not a single microorganism predictive of the risk of NEC. Reasonably, it is the limited diversity of the whole microbiota and the rising abundance of pathogenetic bacteria to be together responsible for the premature susceptibility to NEC (66).

Moreover, a better comprehension in this field is reached considering the influence of gut microbiota on the host immune system.

### The Preterm Lung Microbiota and BPD

A so called “gut-lung axis,” that is a connection between the development of the gut and lung microbiome, has been described (80).

Commensal bacteria in the gut and lungs are necessary for the normal development of the immune homeostasis (80). Therefore, microbial dysbiosis may lead to abnormal inflammatory responses, which are referred to the pathogenesis of BPD.

Airways of the newborn develop during pregnancy influenced by amniotic fluid, placenta, and vagina with its own microbiome (12, 81).

The described lung microbiome at birth evolves over the first weeks and months of postnatal life (82).

Several factors are involved in the development of the lung microbiome. Among them, chorioamnionitis, transplacental infection, or abnormal colonization appear to be able to create an inflammatory process, first step in the pathogenesis of BPD (83). Exposure to prenatal and postnatal antibiotics, respiratory support devices, sepsis, feeding and nutrition, concurrent development of the intestinal microbiome, and the surrounding environmental microbiome, can also be also implicated (82, 84, 85).

In order to support the important role of intrauterine infection as determinant of preterm labor and neonatal diseases, already in 2005 the presence of 16S rRNA was higher in samples of placenta, fetal membranes and cord blood serum from mothers presenting with preterm prelabor rupture of membranes (pPROM) or in spontaneous idiopathic preterm labor, and in the BALF samples or gastric aspirates of their newborns, collected within 24 h of life. BALF samples from these newborns showed a reduction of the prevalence of *Lactobacillus* and a decrease in α-diversity, suggestive for a state of precoscious disbiosys (86).

Most studies described the presence of an airway microbiome early at birth dominated by *Staphylococcus* and *Ureaplasma* (87, 88) and a longitudinal change in the first weeks of life with increasing bacterial loads (34, 35, 89, 90). Lal et al. described the composition of the airway microbiota by analyzing the tracheal aspirates of newborns in the first day of life: a predominance of *Firmicutes* and *Proteobacteria* and the presence of *Actinobacteria, Bacteroidetes, Tenericutes, Fusobacterium, Cyanobacteria*, and *Verrucomicrobia* was observed, without differences between preterm and full term infants (89). Anyway, other authors didn't find adequate bacterial DNA for successful sequence analysis in the tracheal aspirates of 8/10 preterm infants, to sustain the sterility of the airways of intubated preterm infants at birth, with the evidence of a subsequent bacterial colonization after the first 3 days of life (34).

It seems difficult to compare studies about airway microbiome for clinical and methodological heterogeneity. However, the airway colonization pattern appears to be also influenced by lung diseases, particularly by evolution on BPD. In infants who developed BPD compared with those who did not, differences in abundance and a decreased bacterial diversity have been reported (90) (Table 2).

In a study on 94 preterm infants, Wagner et al. found that those who developed severe BPD acquired less initial *Staphylococcus* and high *Ureaplasma* in the first days after birth (90). A possible explanation is that *Ureaplasma* intrauterine exposure downregulates the host response to acute LPS exposure in the preterm sheep model (91). Moreover, host bactericidal activity is related to functional complement and seems to be directly correlated to gestational age (92) (Table 2).

Lohmann and Lal described changes in the relative abundance of *Proteobacteria* and *Firmicutes* but with conflicting data on the preponderance of one or the other (35, 89).

Lohmann et al. (35), at time of intubation, showed a lower bacterial diversity (in terms of lower species count and Shannon diversity index) in newborns who subsequently developed BPD than in those who did not. Concerning the evolution of the lung microbiota, these authors showed a trend to an increase in *Firmicutes* and a decrease in *Proteobacteria* in infants who developed BPD in contrast to the relatively diverse and stable community in the non-BPD group. At the genera level, in both groups *Acinetobacter* was the predominant genus, but its relative abundance decreased longitudinally in the BPD group, while an increasing amount of *Staphylococcus* and *Klebsiella* was observed. Anyway, the changes in bacterial composition do not correlate with the levels of inflammatory cytokines, leaving unanswered the question of the clinical relevance of such findings.

The study of Lal et al. (89) present methodological differences and the airway microbiome of infants after diagnosis of BPD was compared to that of full term newborns matching for post-menstrual age. In contrast to the findings of Lohmann et al., the analysis shows an increase in the phylum *Proteobacteria* and a decrease in the phyla *Firmicutes* and *Fusobacteria* in association with BPD diagnosis. When compared with ELBW newborns and full-term infants, *Gamma Proteobacteria* resulted more abundant whereas *Alpha Proteobacteria* were in lower abundance in BPD infants. At the genus level, the most abundant *Proteobacteria* in BPD patients were *Enterobacteriaceae*.

An interesting finding was the decrease in *Firmicutes* such as *Lactobacillus* in airway microbiome of infants with chorioamnionitis and in preterm infants who went on to develop lung disease (90). This could be an important finding for the association of BPD and chorioamnionitis. Previously, a beneficial role of *Lactobacillus* in other airway diseases has also been reported (93–95).

There are lots of potential determinants of the airway microbiome, but it is difficult to determine whether these changes are causal or established because of a change in treatment or in other factors that lead to BPD.

Even if an association of “antibiotics induced dysbiosis” with BPD has been reported (90), apparently, there are no significant differences in the initial specimen between infants who received empiric antibiotic treatment and those who did not (89). The comparison of the airway microbiome of infants whose mothers received prenatal antibiotics and the infants of mothers who didn't show differences either (90).

The variability in treatments among preterm infants, especially related to antibiotic administration appears to be able to influence these findings (90).

Even if studies which demonstrate a link between dysbiosis and BPD exist, there is a lack in the description of a causal relationship between airway injury during development and respiratory colonization with microorganisms.

Further metabolic analysis of the lung microbiota, including metagenomic, and metabolomic assessments, are necessary to define their role as the cause of lung injury sequence and inflammation.

### The Preterm Gut Microbiota and the Risk of Late Onset Sepsis

It is known that many of the organisms that are responsible for late onset sepsis (LOS) in extremely preterm infants, including *staphylococci*, arise from the intestinal tract (96). Indeed, several studies carried out in extremely preterm infants, have demonstrated the presence of organisms in the feces prior to or concurrent with the onset of LOS which resulted to be caused by the same organism (82, 97, 98).

Puri et al. evaluated infants exposed to intra-amniotic infection and suggested a causative role of specific alterations in the early neonatal microbiome in the development of later sepsis or death (99).

These authors associated an aberrant gastrointestinal colonization with chorioamnionitis or funisitis. The analysis of meconium samples from extremely preterm infants showed, according to the results reported by Moles et al. (100), a definite flora in the first week of life dependent on the exposition to chorioamnionitis or funisitis. A restricted set of taxa was studied in reference to LOS, with a strongest correlation to later sepsis with *Genus Sneathia* (*Phylum Fusobacteria*) and bacteria family (*Phylum Tenericutes*) (99).

Data reported from other authors suggest that in preterm infants is a dysbiosis instead of a predominance of potential pathogens to be associated with sepsis (97) (Table 2). In their case control study, Mai et al. (101) found a predominance of *Firmicutes* in stool samples of newborns with LOS, while Madan et al. (102), by analyzing the early fecal samples of preterm infants with a gestational age between 24 and 27 weeks, highlighted a decreased bacterial diversity with a prevalence of *Proteobacteria* and *Firmicutes* (*Staphylococcus*).

Korpela et al. (31) correlated the risk of sepsis with a disrupted intestinal microbial development in preterm infants, characterized by a predominance of aerobic cocci and a reduction of *bifidobacterial*. In their cohort of preterm newborns, *Enterobacter* was abundant in some sepsis cases, but it was detected also in many cases not developing sepsis.

These data support two possible hypothesis: the translocation across the intestine to blood could be the cause of sepsis or, alternatively, since in some cases the organism responsible of sepsis is not detected in the intestine before the onset of sepsis, it is possible that an intestinal dysbiosis could be responsible for an alteration of the local and systemic immune function (101, 102).

An aberrant immune function could be involved also in the connection between chorioamnionitis, intestinal dysbiosis, and increased susceptibility to LOS. Indeed, a decreased function of the anti-inflammatory T-regulatory cell subset has been found in preterm infants of mother with a diagnosis of chorioamnionitis and/or funisitis (103) (Table 2).

Stewart et al. performed a study showing the association of the gut microbiome and metabolome with the pathogenesis of LOS (104). The gut microbiome of infants with a diagnosis of LOS appeared to be specific for each patient and highly dynamic trough time. Moreover, the identification of one of the most abundant operational taxonomic units in the gut microbiota at diagnosis of LOS with the pathogen in the blood, suggested the translocation trough the gut epithelium as a first element in the pathogenesis of LOS. On the other hand, control infants showed a predominance of *Bifidobacteria*, a taxa that is correlated with some metabolites including raffinose, sucrose, and acetic acid. Among them, a significant role as prebiotic, able to inhibit the growth of potential pathogenic bacteria and to enhance the presence of *Bifidobacterium* spp. (105–107), is conducted by raffinose. It is a α-galactosyl (α-GAL) oligosaccharide that is fermented in the gut by bacteria containing the α-GAL enzyme. While it is reduced in LOS infants before the diagnosis and increased after treatment, in control infants its concentration is constantly high (104).

Neonatal microbiota has been studied also in relation to *GBS* sepsis, which represents a global problem with an estimate overall incidence of about 0.5/1,000 live births. To date, intrapartum antibiotic prophylaxis (IAP) represents a good strategy which lead to a decrement in the incidence of early-onset sepsis (EOS) while LOS rates remained unchanged (108–110).

The route of infection in EOS is well-known, implicating the vertical transmission of *GBS* from the colonized maternal vaginal tract to the infant at birth. In many cases, bacteria entry the respiratory tract trough the aspiration of contaminated fluids, resulting in sepsis or pneumonia during the first days of life (111). The route of infection in LOS is less well-understood. Particularly, it is unknown how the colonization remains stable from the first contact with *GBS* until the disease onset. Also in this case, there are data that show as an aberrant co-development of microbiota and host immunity represent a risk for *GBS* LOS, rather than genetic variations in immune genes alone (112). Affected infants show a decreased presence of anaerobic *Bacteroides* and *Bifidobacterium* spp., while aerobic *Enterobacteria* appear to be increased, when compared to non-septic twin controls (113).

Those observations, together with the evidence of a reduction of intestinal *Bifidobacterium* density in infants whose mothers received IAP, stress how clinicians should be careful in the usage of antibiotics in the early period of life (114). Consistent with this observation, in preterm infants an empirical antibiotic treatment results to be associated to a 3-fold higher risk for LOS caused by various pathogens including *GBS* in preterm infants (115).

Both the depletion of competitive microbes and a delayed immune cell maturation and dysbiosis with the subsequent emergence of pathogenic bacteria, represent a possible way by which antibiotics can influence the microbiome composition.

Recently, concerning the immunological implications, Josefsdottir et al. (116) observed as in antibiotic-treated mice the microbiota is the cause of neutropenia and general depletion of hematopoietic stem cells across multiple lineages. Newborns represent a sensitive population also for its smaller granulocyte pool that may further propagate the negative effects of antibiotics (117), and for the immaturity of phagocytes and adaptive immune cells that reduced the strength to fight against pathogens. Hence, while the adult immune system can respond to a pathogen invasion through the muco-cutaneous barrier, the neonatal immunity may be overwhelmed, resulting in bacterial spread and sepsis.

## Conclusions

The analysis of the knowledge about the preterm infant's microbiota and its relationship with clinical outcomes, show that facultative anaerobes dominated the preterm infant gut, including Enterobacteriaceae, Enterococcus, and Staphylococcus. These are communities that count commonly antibiotic-resistant organisms.

Microbiota plays a key role in the establishment of the gut barrier and many data suggest its immune-modulator role. For all these reasons, the understanding and the prevention of dysbiosis is crucial for the prevention of diseases such as sepsis, NEC and BPD, but may also impact growth rates, immune function, and the risk for various chronic diseases and conditions.

Differences in patient population or care/feeding practices are to take into account in the analysis of the studies conducted in this field. Future studies should be addressed in order to explore differences in the gut/lung microbiota in sub-selected populations, based on specific treatments and probiotic administration. Moreover, concerning the actual acknowledgment about the relation between gut microbiota and the onset of LOS, a deepen description of this link could lead to realized screening approaches able to drive early intervention for the prevention of LOS in the high-risk preterm infants population.

## Author Contributions

CTi, GV, and LP conceptualized and designed the review. CTi and LP analyzed the references material and wrote the manuscript. AP, MT, CA, AL, SD'I, and CTe contributed to the writing of the manuscript. BP, MS, ND, and GV supervised the work and contributed to the writing of the manuscript. All authors reviewed and approved the final version of the manuscript.

### Conflict of Interest

The authors declare that the research was conducted in the absence of any commercial or financial relationships that could be construed as a potential conflict of interest.
